# Prediction of hospital mortality after colorectal perforation surgery from inflammation-based prognostic scores

**DOI:** 10.1016/j.sopen.2022.01.003

**Published:** 2022-01-26

**Authors:** Kensuke Kudou, Tetsuya Kusumoto, Yuho Ebata, Sho Nambara, Yasuo Tsuda, Eiji Kusumoto, Rintaro Yoshida, Yoshihisa Sakaguchi, Koji Ikejiri

**Affiliations:** Department of Gastroenterological Surgery, Clinical Research Institute Cancer Research Division, National Hospital Organization Kyushu Medical Center, Fukuoka, Japan

## Abstract

**Background:**

Inflammation-based prognostic scores have prognostic value in cancer or cardiovascular disease patients. This study evaluated the prognostic value of inflammation-based prognostic scores in colorectal perforation patients.

**Methods:**

Data of 97 patients who underwent surgery for colorectal perforation were reviewed. We calculated various inflammation-based prognostic scores and analyzed the relationship between inflammation-based prognostic score and hospital mortality due to colorectal perforation.

**Results:**

Multivariate analyses of hospital mortality revealed neutrophil–lymphocyte ratio (P = .0021), C-reactive protein/albumin ratio (P = .0224), and prognostic nutritional index (P = .0078) as independent predictive factors. The Kaplan–Meier analysis showed that patients who met all of the following parameters avoided hospital death: neutrophil–lymphocyte ratio < 30, prognostic nutritional index ≥ 27.2, age < 75 years, and perforation of the left colon.

**Conclusion:**

Neutrophil–lymphocyte ratio, C-reactive protein/albumin ratio, and prognostic nutritional index were superior to other inflammation-based prognostic scores in predicting mortality of colorectal perforation. Neutrophil–lymphocyte ratio, prognostic nutritional index, patient's age, and sidedness of the perforation site may be useful parameters to identify subgroups in which a favorable prognosis can be expected.

## Highlights

• NLR, CAR, and PNI are superior in predicting colorectal perforation surgery patients.
• Mortality is associated with high NLR, low PNI, older age, and right-side perforation.
• Favorable prognosis may depend upon NLR, PNI, patient's age, and perforation site side.

## INTRODUCTION

Colorectal perforation has an extremely poor prognosis due to generalized peritonitis and septic shock. As a result of this condition, disseminated intravascular coagulation (DIC) can arise leading to multiple organ failure, resulting in the high mortality rates [1]. In many cases, surgical approaches, including extensive drainage and perioperative intensive treatment with antibiotics, inotropes, polymyxin-B direct hemoperfusion, and respiratory support, are required [2,3]. Despite such aggressive therapy, few patients survive. To improve the prognosis, it is important to assess the condition of patients, choose appropriate surgical procedures, and discern the necessity of perioperative treatment. Previous studies have evaluated several indicators for predicting the prognosis of colorectal perforation [[3], [4], [5], [6], [7]]. Kayano et al showed that a low psoas muscle index is a poor prognostic factor for lower gastrointestinal perforation [3]. Yamamoto et al showed that a high patient age and low preoperative systolic blood pressure were independent risk factors for mortality in patients with colorectal perforation [4]. In addition, scoring systems such as the Acute Physiology and Chronic Health Evaluation II score, physiological and operative severity score for the quantification of mortality and morbidity score, and the Sepsis-related Organ Failure Assessment score were used to investigate the prognostic value of patients who underwent surgery for colorectal perforation [[5], [6], [7]].

On the other hand, inflammation-based prognostic scores (IBPSs), which reflect malnutrition and systemic inflammatory responses, have been reported to predict outcomes in patients with several clinical conditions such as malignant tumors [[8], [9], [10], [11], [12], [13], [14], [15]] and cardiovascular diseases [16,17]. Typical IBPSs include the neutrophil–lymphocyte ratio (NLR), platelet–lymphocyte ratio (PLR), C-reactive protein/albumin ratio (CAR), prognostic nutritional index (PNI), Glasgow Prognostic Score (GPS), and prognostic index (PI). In addition, in patients with gastrointestinal perforation, IBPSs may reflect the degree of peritonitis and systemic inflammation, the time course from the onset, and the nutritional status and may be associated with postoperative prognosis. However, few studies have examined the relationship between the various IBPSs and gastrointestinal perforation. To our knowledge, only one study has demonstrated that NLR and PLR are superior to other IBPSs in predicting the mortality of patients with gastrointestinal perforation [18]. That considered, there are several differences in clinical condition, surgical stress, and survival rates between perforation of the upper and lower gastrointestinal tract. Considering the variation of perforation sites in this study, including the stomach, small intestine, colon, and appendix, we specifically analyzed patients who underwent surgery for colorectal perforation and evaluated the prognostic value of IBPSs such as NLR, PLR, CAR, PNI, GPS, and PI. Moreover, we suggested a new score based on IBPSs and routinely available parameters to identify subgroups in which a favorable prognosis can be expected following surgery.

## METHODS

### Patients

In this retrospective, single-center, cohort study, we reviewed the data of 105 patients who underwent emergency surgery for colorectal perforation at our institute between August 2010 and December 2019. Among the 105 patients, 8 patients whose clinical data were incomplete were excluded. Finally, data of 97 patients who underwent surgery for colorectal perforation were eligible for analysis. The patients were divided into two groups according to hospital mortality: survivors and nonsurvivors.

Permission to perform this study was provided by the Institutional Review Board of the National Kyushu Medical Center (20C033). The study conforms to the provisions of the Declaration of Helsinki (as revised in Fortaleza, Brazil, October 2013), available at https://www.wma.net/what-we-do/medical-ethics/declaration-of-helsinki/.

### IBPSs

**Fig 1:**
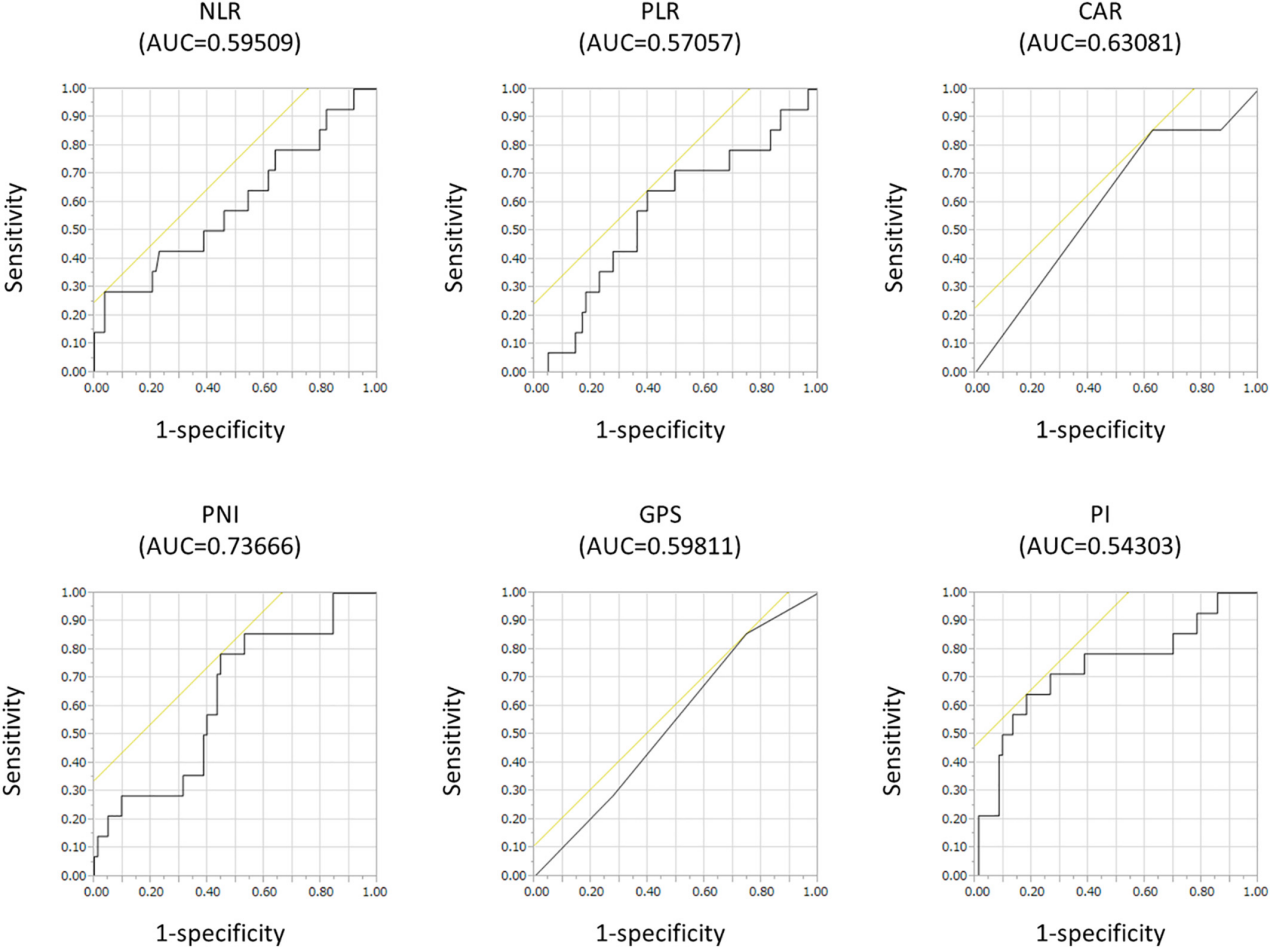
Comparison of the predictive ability of 6 inflammation-based prognostic scores—NLR, PLR, CAR, PNI, GPS, and PI—by receiver operating characteristic curve analyses.

The NLR, PLR, CAR, PNI, GPS, and PI were calculated from the patient records. Baseline blood data were obtained by collecting blood from the peripheral vein of each patient before surgery. NLR and PLR were defined as absolute neutrophil count and platelet count, respectively, divided by the absolute lymphocyte count [10,11]. CAR was defined as the serum CRP level divided by the serum albumin level [12]. The PNI was calculated using the following formula: 10 × serum albumin (g/dL) + 0.005 × total lymphocyte count (per mm^3^) [13]. The GPS was calculated by CRP and albumin using standard thresholds (> 1.0 mg/dL for CRP and < 3.5 g/dL for albumin) [14]. Calculation of the PI was based on the CRP level and white blood cell count. The upper limits of reference ranges for the CRP level (0.1 mg/dL) and the white blood cell count (11,000/mm^3^) were used as cutoff values [15]. The PI was considered as 0 if both values were less than the cutoff values, and the PI was 1 if one of the two markers was elevated (Table 1). Receiver operating characteristic (ROC) curve analysis was used to identify the optimal cutoff values of these IBPSs (Fig. 1).

**Table 1:** Baseline demographic and clinical features of survivors and nonsurvivors with colorectal perforation

*Factor*		*Survivors (*n *= 83),* n *(%)*	*Nonsurvivors (*n *= 14),* n *(%)*	P *value*
Sex	Male	44 (53.0)	5 (35.7)	.2615
	Female	39 (47.0)	9 (64.3)	
Age in years	Mean	69.8 ± 1.4	77.9 ± 3.4	.0312
	(Range)	(26–95)	(64–91)	
Location	C	2 (2.4)	3 (21.4)	
	A	4 (4.8)	2 (14.3)	
	T	8 (9.6)	1 (7.1)	
	D	5 (6.0)	0 (0.0)	
	S	49 (59.0)	7 (50.0)	
	R	15 (18.1)	1 (7.1)	
Laterality of perforation site	Right	14 (16.9)	6 (42.9)	.0370
	Left	69 (83.1)	8 (57.1)	
Causes of perforation	Diverticulum	29 (34.9)	1 (7.1)	
	Cancer	20 (24.1)	5 (35.7)	
	Post-ESD or EMR	7 (8.4)	0 (0.0)	
	Steroid	3 (3.6)	1 (7.1)	
	Others	13 (15.7)	4 (28.6)	
	Unknown	11 (13.3)	2 (14.3)	
Chemotherapy at the time of perforation in patients with cancer (*n* = 25)	No	14 (70.0)	3 (60.0)	1.0000
	Yes	6 (30.0)	2 (40.0)	
NLR	Mean	10.4 ± 1.4	19.6 ± 3.4	.0155
	(Range)	(0.9–40.8)	(1.6–97)	
PLR	Mean	397.2 ± 35.9	420.2 ± 87.4	.8083
	(Range)	(36.9–2137)	(89–1126)	
CAR	Mean	4.6 ± 0.6	7.5 ± 1.5	.0736
	(Range)	(0.0–19.2)	(0.1–23.3)	
PNI	Mean	34.7 ± 0.9	27.7 ± 2.2	.0051
	(Range)	(16.2–65.0)	(18.5–42.7)	
GPS	0/1	31 (37.4)	2 (14.3)	.1294
	2	52 (62.7)	12 (85.7)	
PI	0	21 (25.3)	2 (14.3)	.5078
	1/2	62 (74.7)	12 (85.7)	

Data are presented as number (%), unless otherwise stated. *C*, cecum; *A*, ascending colon; *T, transverse colon; D, descending colon; S, sigmoid colon; R, rectum; ESD, endoscopic submucosal dissection; EMR, endoscopic mucosal resection.*

### Hospital Mortality and IBPSs

The cause of colorectal perforation, especially the presence of cancer, affects long-term outcomes. Therefore, we discerned that hospital mortality was the most suitable parameter for evaluating the postoperative prognosis of colorectal perforation and applied it for prognosis analysis.

To evaluate the relationship between hospital mortality of colorectal perforation and each biomarker, including IBPSs, univariate and multivariate analyses with a Cox proportional hazard model were performed.

### Statistical Analysis

Differences in characteristics between the groups were evaluated using Fisher's Exact Test or unpaired *t* test. Survival curves were plotted according to the Kaplan–Meier method, and differences were analyzed using the log-rank test. Univariate and multivariate analyses were performed using a Cox proportional hazard model to identify independent prognostic factors. All *P* values were 2-sided. ROC curve analysis was used to determine the predictive value of the combined index. All analyses were performed using JMP PRO 11 software (SAS Institute Inc, https://www.jmp.com/ja_jp/home.html).

## RESULTS

### Characteristics of Patients with Colorectal Perforation

The patients were divided into 2 groups according to hospital mortality; 83 (86%) patients were survivors, and 14 (14%) were nonsurvivors. The baseline characteristics of the survivors and nonsurvivors are summarized in Table 1. The mean age was 69.8 (range, 26–95) years for survivors and 77.9 (range, 64–91) years for nonsurvivors (*P* = .0312). Regarding the location of perforation, the sigmoid colon was the most frequent in both groups (survivors, 59.0%; nonsurvivors, 50.0%). The incidence of perforation in the right-sided colon was significantly higher in nonsurvivors than in survivors (survivors, 16.9%; nonsurvivors, 42.9%; *P* = .0370). Diverticula were the most common cause of perforation in survivors (34.9%), whereas cancer was the most frequent cause (35.7%) in nonsurvivors. Comparing the mean values of each IBPS between survivors and nonsurvivors, NLR and PNI were significantly worse in nonsurvivors than in survivors (*P* = .0155 and .0051, respectively) (Table 1).

Among 25 patients with cancer, only 8 were undergoing systemic chemotherapy at the time of perforation. A comparison of clinical features according to chemotherapy before the onset of perforation showed no significant differences in the mean values of each IBPS between the 2 groups (Supplementary Table 1).

### Operative Outcomes

Operative outcomes were compared between survivors and nonsurvivors (Table 2). There were no significant differences in mean operative times (150.0 vs 143.1 minutes, *P* = .6673) and intraoperative blood loss (122.9 vs 99.9 mL, *P* = .6996) between the 2 groups. The degree of postoperative complications was categorized according to the Clavien–Dindo (C-D) classification [19,20]. The occurrence of all C-D grades of postoperative complications was higher in nonsurvivors than in survivors (survivors, 48.2% vs nonsurvivors, 78.6%; *P* = .0445). The ratio of severe complications (C-D grade ≥ IIIa) was more remarkable in nonsurvivors (survivors, 13.3% vs nonsurvivors, 71.4%; *P* < .0001). The median survival time of nonsurvivors was 18.5 days.

**Table 2:** Comparison of operative outcomes between survivors and nonsurvivors with colorectal perforation

*Factor*		*Survivors (*n *= 83),* n *(%)*	*Nonsurvivors (*n *= 14),* n *(%)*	P *value*
Surgical procedure	Stoma	40 (48.2)	5 (35.7)	
	Resection + anastomosis	6 (7.2)	1 (7.1)	
	Resection + anastomosis + stoma	3 (3.6)	1 (7.1)	
	Resection + stoma	33 (39.8)	7 (50.0)	
	Others	1 (1.2)	0 (0.0)	
Surgical approach	Open	47 (56.6)	9 (64.3)	.7716
	Laparoscopic	36 (43.4)	5 (35.7)	
Operative time (min)	Mean	150.0 ± 6.0	143.1 ± 14.6	.6673
	(Range)	(35–298)	(41–233)	
Intraoperative blood loss (mL)	Mean	122.9 ± 22.6	99.9 ± 55.1	.6996
	(Range)	(0–1032)	(0–365)	
Postoperative complication	No	43 (51.8)	3 (21.4)	.0445
	Yes	40 (48.2)	11 (78.6)	
Surgical site infection	No	76 (91.6)	13 (92.9)	1.0000
	Yes	7 (8.4)	1 (7.1)	
Intra-abdominal abscess	No	74 (89.2)	13 (92.9)	1.0000
	Yes	9 (10.8)	1 (7.1)	
Ileus	No	75 (90.4)	14 (100.0)	.5978
	Yes	8 (9.6)	0 (0.0)	
CD grade ≥ 3a	< 3a	72 (86.7)	4 (28.6)	<.0001
	≥ 3a	11 (13.3)	10 (71.4)	
Hospital stay (d)	Mean	49.0 ± 4.0	43.1 ± 9.8	.5766
	(Range)	(12–232)	(1–108)	
Median survival time (d)		1590	18.5	

Data are presented as number (%), unless otherwise stated.

### Prognostic Factors for Hospital Mortality

Based on ROC curve analysis, the optimal cutoff values of the NLR, PLR, CAR, PNI, GPS, and PI were identified as 30, 365, 2.8, 27.2, 2, and 1, respectively, and the area under the curve values were 0.59509, 0.57057, 0.63081, 0.73666, 0.59811, and 0.54303, respectively (Fig 1).

Univariate analyses revealed that perforation of the right side of the colon, postoperative complications, NLR ≥ 30, CAR ≥ 2.8, and PNI < 27.2 were associated with postoperative hospital mortality in patients with colorectal perforation. Multivariate analyses revealed that NLR ≥ 30 (hazard ratio [HR] = 11.18, *P* = .0021), CAR ≥ 2.8 (HR = 3.930, *P* = .0224), and PNI < 27.2 (HR = 4.400, *P* = .0078) were independent predictive factors for hospital mortality in patients with colorectal perforation (Table 3).

**Table 3:** Univariate and multivariate analyses for hospital mortality of colorectal perforation

*Factor*	*Univariate analysis*			*Multivariate analysis*			*Multivariate analysis*			*Multivariate analysis*			*Multivariate*			*Multivariate analysis*			*Multivariate analysis*		
	*HR*	*(95% CI)*	P *value*	*HR*	*(95% CI)*	P *value*	*HR*	*(95% CI)*	P *value*	*HR*	*(95% CI)*	P *value*	*HR*	*(95% CI)*	P *value*	*HR*	*(95% CI)*	P *value*	*HR*	*(95% CI)*	P *value*
Age ≥ 75 y (vs < 75 y)	2.354 (0.812–7.665) .1154			1.938 (0.651–6.459) .2373			2.005 (0.682–6.608) .2084			2.142 (0.710–7.312) .1787			1.780 (0.577–6.080) .3186			2.232 (0.748–7.555) .1521			2.493 (0.823–8.498) .1070		
Right side of colon (vs. left)	3.095 (1.018–8.902) .0466			5.482 (1.562–21.50) *.0086*			2.718 (0.872–8.043) .0826			2.802 (0.906–8.222) .0720			2.577 (0.835–7.543) .0965			3.061 (0.994–8.969) .0512			3.256 (1.057–9.551) .0404		
Postoperative complication	3.577 (1.116–15.82) *.0309*			2.698 (0.802–12.30) .1131			2.959 (0.896–13.30) .0768			2.711 (0.789–12.45) .1173			2.871 (0.850–13.14) .0921			2.796 (0.832–12.67) .0994			2.801 (0.839–12.65) .0969		
Cancer (versus others)	1.777 (0.545–5.158) .3195			1.586 (0.480–4.691) .4267			1.588 (0.483–4.651) .4235			2.004 (0.597–6.092) .2450			1.929 (0.578–5.811) .2680			1.881 (0.565–5.629) .2850			1.871 (0.559–5.646) .2915		
NLR ≥ 30 (vs < 30)	6.907 (1.882–20.86) *.0060*			11.18 (2.594–48.21) *.0021*			–			–			–			–			–		
PLR ≥ 365 (vs < 365)	1.920 (0.667–5.836) .2244			–			1.482 (0.496–4.633) .4785			–			–			–			–		
CAR ≥ 2.8 (vs < 2.8)	4.150 (1.295–18.35) *.0152*			–			–			3.930 (1.200–17.62) .0224			–			–			–		
PNI < 27.2 (vs ≥ 27.2)	5.243 (1.820–15.95) *.0026*			–			–			–			4.400 (1.493–13.62) *.0078*			–			–		
GPS = 2 (vs < 2)	3.415 (0.930–21.95) .0660			–			–			–			–			3.517 (0.939–22.92) .0635			–		
PI ≥ 1 (vs < 1)	1.977 (0.539–12.71) .3344			–			–			–			–			–			2.740 (0.694–18.48) .1632		

*P* values in italic are statistically significant.

## Postoperative Prognosis Based on the IBPSs

**Fig 2:**
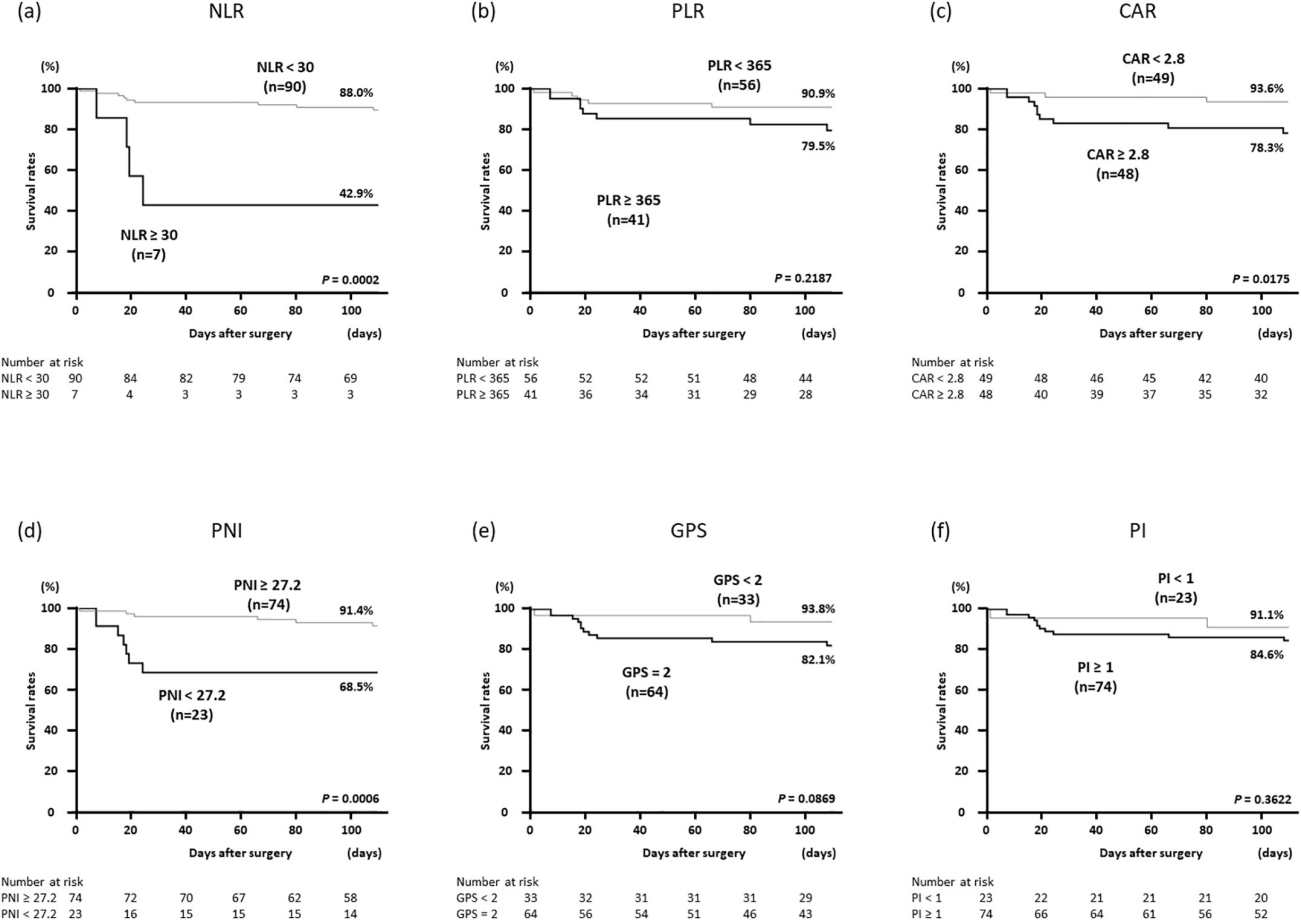
Postoperative hospital mortality in patients with colorectal perforation based on 6 inflammation-based prognostic scores such as NLR, PLR, CAR, PNI, GPS, and PI. The Kaplan–Meier method was performed according to each cutoff value. The optimal cutoff values of NLR, PLR, CAR, PNI, GPS, and PI were determined to be 30, 365, 2.8, 27.2, 2, and 1, respectively.

Patients were divided into 2 groups according to the cutoff values of each IBPS, and the Kaplan–Meier method was used to compare the hospital mortality between the groups. These results showed that patients with NLR ≥ 30 (vs NLR < 30) (survival rates: 42.9% vs 88.0%, *P* = .0002), CAR ≥ 2.8 (vs CAR < 2.8) (survival rates: 78.3% vs 93.6%, *P* = .0175), and PNI < 27.2 (vs PNI ≥ 27.2) (survival rates: 68.5% vs 91.4%, *P* = .0006) were significantly associated with higher hospital mortality after surgery for colorectal perforation (Fig 2).

### New Scoring

**Fig 3:**
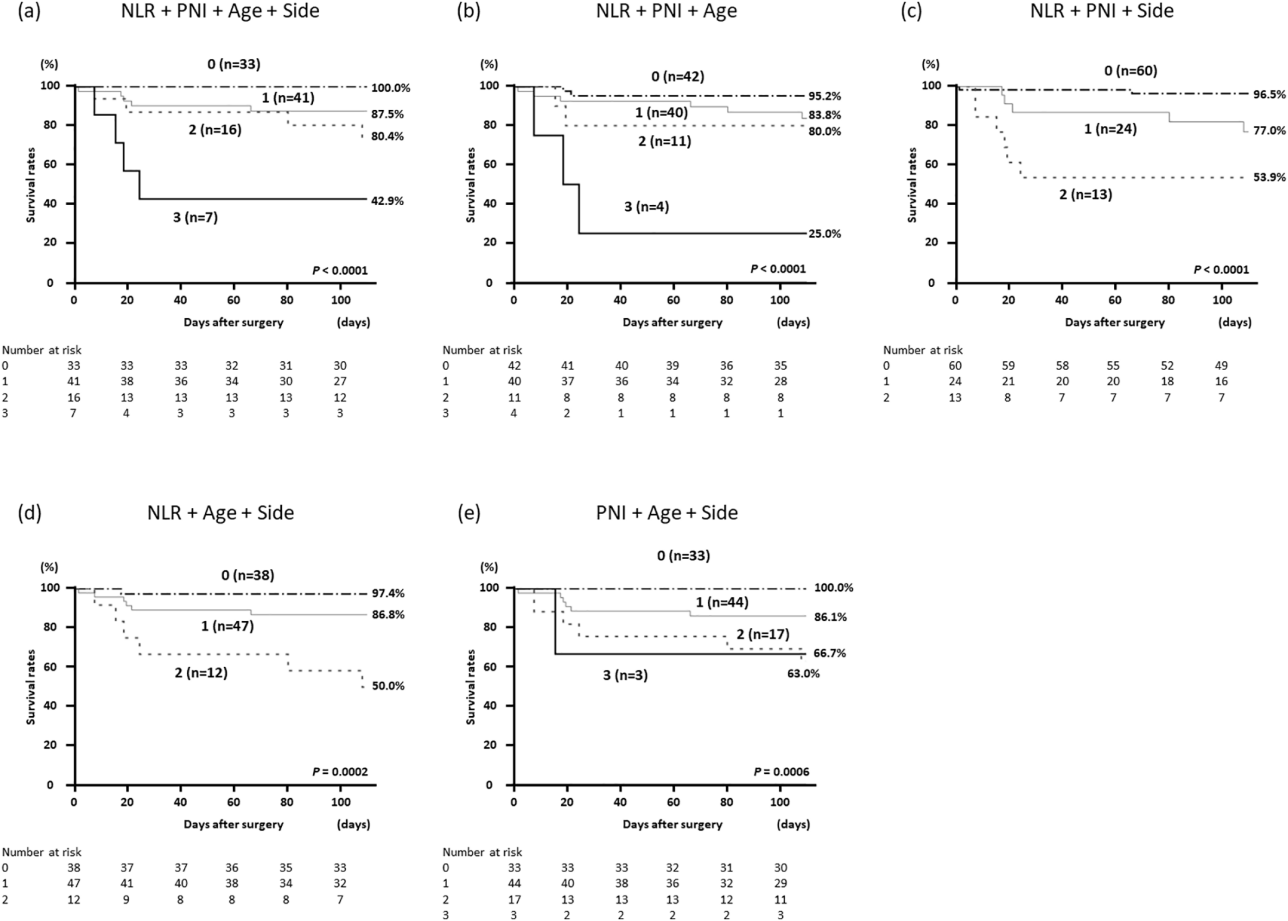
Postoperative hospital mortality in patients with colorectal perforation based on the new scoring system comprising NLR, PLR, age, and sidedness of perforation site. The new score was calculated according to Table 4. New score consisting of (A) all 4 parameters, (B) 3 parameters except sidedness, (C) 3 parameters except age, (D) 3 parameters except PNI, and (E) 3 parameters except NLR, respectively.

When patients were categorized into survivors and nonsurvivors, there were significant differences in age, sidedness of the perforation site, and mean values of NLR and PNI (Table 1). Based on these results, we regarded NLR ≥ 30, PNI < 27.2, age ≥ 75 years, and perforation of the right side of the colon as risk factors for predicting prognosis and established a new score by combining these 4 parameters (Table 4). The Kaplan–Meier curves according to the new score showed that the survival rates of patients with higher scores were significantly poor, whereas 100% of patients who met all of the following 4 parameters avoided hospital death: NLR < 30, PNI ≥ 27.2, age < 75 years, and perforation of the left colon (Fig 3, *A*). However, when any one of PNI, age, or sidedness was lacking from the new score, survival rates were not 100% even in patients with a score of 0 (Fig 3, *B–E*).

**Table 4:** Calculation of the new score

*Factor*		*Score*
NLR	< 30	0
	≥ 30	1
PNI	≥ 27.2	0
	< 27.2	1
Age (y)	< 75	0
	≥ 75	1
Sidedness	Left	0
	Right	1
New score	(= total sum of each score of the four scores)	0 to 4

## DISCUSSION

In this study, we evaluated the prognostic significance of various IBPSs in patients who underwent surgery for colorectal perforation and demonstrated that NLR, CAR, and PNI were superior to the other IBPSs in predicting mortality. Moreover, patients with NLR < 30, PNI ≥ 27.2, age < 75 years, and perforation of the left colon were identified as the subgroup in which a better prognosis could be expected.

IBPSs have been reported as predictive factors for the prognosis of various malignant tumors in previous studies. These biomarkers can be evaluated easily by collecting blood from a peripheral vein, and the methods of calculation are simple. IBPSs reflect systemic inflammatory response, immunosuppression, malnutrition, or cachexia due to tumor progression [21]. In addition, a small number of studies have reported that several IBPSs were correlated with prognosis in cases of nonmalignant diseases such as acute pancreatitis, pulmonary embolism, and infective endocarditis [[22], [23], [24]]. In these studies, NLR and PNI were mainly applied as predictive factors, which were consistent with our results. NLR is elevated by reflecting neutrophilia and lymphopenia with systemic inflammation. The neutrophil count increases rapidly during acute systemic inflammation, which may occur due to peritonitis and sepsis. In contrast, lymphopenia reflects immunosuppression. Colorectal perforation can cause peritonitis, intra-abdominal abscesses, and septic shock. These complications affect the failure to rescue patients. The condition of immunosuppression reflecting lymphopenia may be a lethal factor when these complications occur. Therefore, NLR may be an important prognostic factor not only in the condition of malignant diseases but also in acute systemic inflammation or infectious diseases.

The PNI value is determined by the serum albumin and total lymphocyte count. Serum albumin level is an indicator of nutritional status. Hypoalbuminemia may occur due to a systemic inflammatory response. Several studies have compared the validity of multiple IBPSs to predict the prognosis of gastrointestinal malignancies [9,21,25,26], and most reports indicated that PNI was a useful prognostic factor that was superior to other IBPSs. Many studies, including ours, suggest that PNI is a versatile prognostic factor, and a low PNI value indicates an unfavorable condition in various diseases.

In the present study, several routinely available parameters other than IBPS were also compared between survivors and nonsurvivors. We focused on patients' age and laterality of the perforation site because the mean age and the incidence of perforation in the right-side colon were significantly higher in nonsurvivors (*P* = .0312 and .0370, respectively; Table 1). It is presumed that patients of higher age are likely to have more comorbidities that affect the postoperative recovery and they have fewer reserve capacities to withstand peritonitis and surgical stress than younger patients. Regarding laterality, it is known that the gut microbiota between the right and left colon is different. *Prevotella*, *Selenomonas*, and *Peptostreptococcus* were identified in relatively higher abundances in right-side colon, while *Fusobacterium*, *Escherichia*/*Shigella*, and *Leptotrichia* were relatively abundant in left-side colon among colorectal cancer patients [27]. However, the relationship between these differences in the gut microbiota and the prognosis of colorectal perforation remains unresolved. One conceivable reason why right-sidedness is associated with a poorer prognosis is that perforation of the right colon leads to a higher risk of widespread peritonitis due to unconsolidated stool than perforation of the left colon and rectum. Although only 1 or some IBPSs cannot perfectly predict survival in patients with colorectal perforation, our analysis summarized in Figure 3 indicates that the addition of these 2 parameters, namely, patient's age and sidedness of the colonic perforation, may be able to identify subgroups with better prognosis. In the clinical setting, this new score may be useful for accurate prediction of fatal disease. This would help guide the decision on the need for earlier hospice care after surgery and may aid in determining the specific guidance and counseling offered to patients and their families.

However, this study has a potential limitation. This was a retrospective, single-institution study. However, colorectal perforation occurs suddenly and usually requires emergency surgery. Therefore, designing a prospective study on colorectal perforation is difficult. Few reports have demonstrated the relationship between IBPS and outcomes after surgery for colorectal perforation. Thus, the accumulation of findings from retrospective studies is valuable.

In conclusion, in various inflammation-based prognostic scores, NLR, CAR, and PNI were superior to other IBPSs in predicting mortality in patients who underwent surgery for colorectal perforation. Patients with NLR < 30, PNI ≥ 27.2, age < 75 years, and perforation of the left colon were identified as subgroups in which a favorable prognosis could be expected.

The following are the supplementary data related to this article.

Supplementary Table 1 Baseline demographic and clinical features of cancer patients in the 2 groups according to the presence of systemic chemotherapy at the time of colorectal perforation Supplementary Table 1
