# Supplementary material for: Prediction of hospital mortality after colorectal perforation surgery from inflammation-based prognostic scores
Source: Surg Open Sci. 2022 Jan 26;8:40–6. doi: 10.1016/j.sopen.2022.01.003 (PMC8914212; doi:10.1016/j.sopen.2022.01.003)
Supplement: Supplementary Table 1 — Baseline demographic and clinical features of cancer patients in the 2 groups according to the presence of systemic chemotherapy at the time of colorectal perforation [file mmc1.docx]

**Supplementary Table 1.** Baseline demographic and clinical features of cancer patients in the two groups according to the presence of systemic chemotherapy at the time of colorectal perforation

| Factor |  | No  (n = 17)  n (%) | Yes  (n = 8)  n (%) | P-value |
| --- | --- | --- | --- | --- |
| Sex | Male  Female | 8 (52.9)  9 (47.1) | 4 (50.0)  4 (50.0) | 1.0000 |
| Age in years | Mean  (range) | 71.2 ± 3.6  (26-92) | 63.5 ± 5.3  (47-71) | 0.2427 |
| Location | C  A  T  D  S  R | 2 (11.8)  0 (0.0)  1 (5.9)  1 (5.9)  9 (52.9)  4 (23.5) | 0 (0.0)  2 (25.0)  2 (25.0)  1 (12.5)  3 (37.5)  0 (0.0) |  |
| Laterality of perforation site | Right  Left | 3 (17.6)  14 (82.4) | 4 (50.0)  4 (50.0) | 0.1563 |
| NLR | Mean  (range) | 11.8 ± 2.4  (1.5-39.5) | 8.4 ± 3.5  (2.8-16.9) | 0.4258 |
| PLR | Mean  (range) | 420.0 ± 51.0  (134.6-865.5) | 304.7 ± 74.4  (89.0-485.8) | 0.2140 |
| CAR | Mean  (range) | 6.2 ± 1.6  (0.1-18.8) | 5.6 ± 2.3  (0.3-19.2) | 0.8198 |
| PNI | Mean  (range) | 31.5 ± 1.7  (18.7-43.1) | 33.0 ± 2.5  (24.8-43.3) | 0.6249 |
| GPS | 0/1  2 | 6 (35.3)  11 (64.7) | 2 (25.0)  6 (75.0) | 1.0000 |
| PI | 0  1/2 | 5 (29.4)  12 (70.6) | 1 (12.5)  7 (87.5) | 0.6237 |

Data are presented as number (%), unless otherwise stated. C, Cecum; A, ascending colon

T, transverse colon; D, descending colon; S, sigmoid colon; R, rectum; ESD, endoscopic submucosal dissection; EMR, endoscopic mucosal resection; NLR, neutrophil-lymphocyte ratio; PLR, platelet-lymphocyte ratio; CAR, C-reactive protein/albumin ratio; PNI, prognostic nutritional index; GPS, Glasgow prognostic score; PI, prognostic index.
